# Atherosclerotic Lesion of the Carotid Artery in Indonesian Cynomolgus Monkeys Receiving a Locally Sourced Atherogenic Diet

**DOI:** 10.3390/vetsci9030105

**Published:** 2022-02-26

**Authors:** Sri Rahmatul Laila, Dewi Apri Astuti, Irma Herawati Suparto, Ekowati Handharyani, Thomas C. Register, Dondin Sajuthi

**Affiliations:** 1 Departement of Anatomy, Physiology, and Pharmacology, Faculty of Veterinary Medicine, IPB University, Bogor 16680, Indonesia; 2 Departement of Nutrition and Feed Technology, Faculty of Animal Science, IPB University, Bogor 16680, Indonesia; dewiaa@apps.ipb.ac.id; 3 Primate Research Center, IPB University, Bogor 16680, Indonesia; irmasu@apps.ipb.ac.id (I.H.S.); ekowatieko@apps.ipb.ac.id (E.H.); dondinsa@apps.ipb.ac.id (D.S.); 4 Departement of Chemistry, Faculty of Mathematics and Natural Sciences, IPB University, Bogor 16680, Indonesia; 5 Departement of Veterinary Clinic, Reproduction, and Pathology, Faculty of Veterinary Medicine, IPB University, Bogor 16680, Indonesia; 6 Centre for Comparative Medicine Research, Wake Forest School of Medicine, Winston-Salem, NC 27157, USA; register@wakehealth.edu

**Keywords:** atherosclerosis, carotid artery, CD68, cynomolgus monkey, lesions, diet

## Abstract

The atherosclerotic lesion is a principal hallmark of atherosclerotic animal models. This study aimed to assess lesions of the carotid artery in Indonesian cynomolgus monkeys exposed to an IPB-1 atherogenic diet. A total of 20 adult male cynomolgus monkeys received the local IPB-1 diet for two years. Blood lipid profiles, morphology, and carotid ultrasound of monkeys were measured. Nine of them were euthanized to confirm atherosclerotic lesions. Common carotid arteries (CCA) and carotid bifurcation (BIF) samples were collected and stained using Verhoef-van Giessen and CD68 immunohistochemistry. The results reveal the presence of severe atherosclerosis plaques in six out of nine animals (66.7%) corresponding to intermediately and hyper-responsive groups. The hyper-responsive group displayed the highest response in the developing intimal area (IA) at the CCA (0.821 mm^2^), whereas the hyporesponsive group had the smallest IA (0.045 mm^2^) (*p* = 0.0001). At the BIF, the hyporesponsive group showed the smallest IA (*p* = 0.001), but there was no difference between the intermediately and hyper-responsive groups (*p* = 0.312). The macrophage marker CD68 was also expressed on the cartotid of the intermediately and hyper-responsive groups. These results indicate that severe atherosclerotic lesions with high infiltration of macrophages were formed in the carotid arteries of intermediately and hyper-responsive Indonesian cynomolgus monkeys fed with the local atherogenic diet IPB-1 over two years, thus confirming atherosclerosis in a nonhuman primate model.

## 1. Introduction

The cynomolgus monkey (*Macaca fascicularis*) is the non-human primate most used in biomedical research after the rhesus monkey (*Macaca mulatta*). Indonesian cynomolgus monkeys are exported primarily to the United States [1] for biomedical research pertaining to infectious diseases as well as degenerative diseases [2]. The Indonesian cynomolgus monkey (*M. fascicularis fascicularis*) has a unique genetic profile, which makes it particularly suitable for biomedical research [3]. Cynomolgus is used in biomedical research, with many advantages including anatomical and physiological aspects that are similar to humans, such as in cardiovascular disease [4]. In atherosclerosis, cynomolgus can be used to study the mechanisms of pathophysiology and the formation of lesions, up to the appearance of clinical symptoms due to such lesions [5]. However, the International Union for Conservation of Nature (IUCN) has reported that the cynomolgus population has significantly decreased, making its status vulnerable [6], which raises awareness regarding the use of cynomolgus monkeys in research.

In the last decade, our team at the Bogor Primate Research Center (PRC)-IPB University, Indonesia, has lead an initiative promoting biomedical research with cynomolgus monkeys nationally to reduce exportation and provide early treatment prior to advance studies abroad. This initiative resulted in the development of a local diet for atherosclerosis studies named the Institut Pertanian Bogor-1 (IPB-1) atherogenic diet. This diet had good digestibility value and is well absorbed, allowing the maintenance of normal body weight and adipose index of the monkeys [7]. This diet can also be used for long-term feeding without inducing major complications in animals [8]. In a previous study, the IPB-1 atherogenic diet was able to induce hypercholesterolemia and increase the intima–media thickness surrounding the carotid artery, as clinically observed by ultrasound, in cynomolgus monkeys [9]. These results support conducting clinical research on atherosclerosis lesions without euthanizing the monkeys. However, further observations characterizing the formation of atherosclerotic lesions in the carotid artery are warranted for confirmation that cynomolgus monkeys receiving the IPB-1 diet represent an excellent model for investigating human atherosclerotic lesions of the artery.

The atherosclerotic lesion is one of the main parameters that are still widely used for evaluating atherosclerosis in animal models. As an indicator of atherogenesis, cellular and biochemical components can be detected by histological techniques. The initiation process of atherosclerosis involves the trapping of low-density lipoprotein (LDL) in the intimal matrix and its oxidation, together with the stimulation of inflammatory and immune responses that are associated with intimal migration and/or proliferation of particular cell types, predominantly macrophages [10,11]. The development of an atherosclerosis lesion begins with endothelial dysfunction, a fatty streak, atheroma, followed by a fibrous cap atheroma, and finally complex plaques [12]. Previous studies, which were mainly conducted in the United States using a “Western diet”, have reported atherosclerotic lesions in cynomolgus monkeys since the 1970s [13,14,15,16]. The IPB-1 atherogenic diet differs from the Western diet in terms of composition and nutrition, making it relevant to thoroughly test its outcomes on the atherosclerotic lesion, which is key to developing an Indonesian monkey model for atherosclerosis. Our previous findings demonstrate 3 response groups including the slightly elevated cholesterol in the plasma of monkeys (intermediate responsive group) [9], while Clarkson et al. only observed this for the hypo- and hyper-responsive groups [14]. It is interesting to note that atherosclerotic lesions in the carotid artery of individuals in the intermediately responsive group would be the same as those in the hyper-responsive group. In this study, we evaluated the grade of the plaques (American Heart Association/AHA grade) [17,18] and a cellular marker of macrophages involved in atherogenesis [19]. The establishment of the IPB-1-diet-induced monkey model could help researchers to perform studies on the prevention, detection, and treatment of atherosclerosis diseases in their country of origin.

## 2. Materials and Methods

### 2.1. Experimental Design

We used a locally sourced diet, the IPB-1 atherogenic diet, produced at PRC-IPB, Indonesia. This diet contains egg yolk as a cholesterol source, coconut oil and beef tallow as a saturated fatty acid, and corn oil as a polyunsaturated fatty acid (PUFA) [7]. The macronutrient differences between the IPB-1 atherogenic diet and the Western diet formula for the cynomolgus model [14] are shown in Table 1. All procedures were approved by the Animal Care and Use Committee from PRC-IPB (Number PRC-14-B003). A total of 20 male cynomolgus monkeys (aged 6–7 years, weight 5–6 kg) were fed the IPB-1 atherogenic diet (containing 0.28 mg/Cal of cholesterol daily) for two years. We previously measured different atherosclerosis risk factors (total plasma cholesterol (TPC), LDL, high-density lipoprotein (HDL), triglyceride (TG), blood glucose (Glu), and body mass index (BMI)) in all of the monkeys included in the current study [9]. These monkeys showed different responses to the cholesterol content of the diet. The differential response of cynomolgus monkeys to dietary cholesterol was reported in another study previously in which monkeys were divided only into 2 groups, hyporesponsive, for TPC around 200 mg/dL, and hyper-responsive, for TPC above 400 mg/dL [14]. In this current study, we observed monkeys with TPC between 200 and 400 mg/dL, so the monkeys were divided into three groups: hyporesponsive (TPC < 200 mg/dL), intermediately responsive (TPC 200–400 mg/dL), and hyper-responsive (TPC > 400 mg/dL) groups, corresponding to four, eight, and eight monkeys in each group, respectively. For post-mortem investigation, we euthanized nine monkeys using a group randomized design, in which three monkeys represented each group (hypo-, intermediately, and hyper-responsive).

### 2.2. Necropsy Procedure

The necropsy was performed by a certified pathologist following the procedure in the Laboratory of Pathology, PRC-IPB, Bogor. Ketamine 10% (Ket-A-100^®^, Senasa, Peru) 10 mg/kg of body weight (BW) and xylazine 2% (Dormi-Xyl^®^ 20, Senasa, Peru) 0.5 mg/kg of BW were administered intramuscularly as a sedative agent before necropsy. Afterwards, a low dose of sodium pentobarbital (Doléthal^®^, Espanola) (100 mg/kg) was injected intravenously to prevent sudden death of the monkeys. The heart of the monkey was kept beating for the transcardial perfusion procedure. The circulatory system was flushed with NaCl saline (0.9%) and perfused with 4% paraformaldehyde for 1 h under a pressure of 100 mm Hg [20]. The common carotid arteries (CCA) and the carotid bifurcation (BIF) were collected at necropsy and post-fixed into 4% paraformaldehyde for about 48 h (4 °C). One block was taken from the BIF, while one every three blocks were taken from the right and left CCA (5 mm in length, cut perpendicular to the long axis of the artery) then stored at a specific tissue cassette in stopping point alcohol 70%.

### 2.3. Histological Preparation and Verhoef-Van Gieson (VVG) Staining

All the selected artery blocks were dehydrated in graded alcohol (80, 90, 95, and 100%), cleared with three changes of xylol, then infiltrated and embedded in paraffin (20). The paraffin blocks of carotid arteries were cut to 0.5 µm thickness using a manual rotary microtome. From each block, two serial sections were mounted, one on a non-coated slide for VVG histochemical staining, and one on a poly-L-lysine coated slide for CD-68 /macrophages immunohistochemical (IHC) staining.

VVG staining was performed to identify elastic fibers of the carotid arteries, using a protocol provided by the pathology laboratory, Section for Comparative Medicine, Wake Forest University School of Medicine, Winston-Salem, NC, USA. The slides were first deparaffinized and hydrated with distilled water. They were subsequently stained in Verhoeff’s solution for 1 h, and then rinsed in tap water with 2–3 changes. Next, the slides were differentiated in 2% ferric chloride for 1–2 min then rinsed with tap water to stop the differentiation and examined microscopically (light microscope with 10× objective lens magnification) for black elastic fiber staining and gray background. The next step was to treat the slides with 5% sodium thiosulfate for 1 min followed by washing in running tap water for 5 min. Furthermore, the slides were counterstained in Van Gieson’s solution for 3–5 min, followed by dehydration through 95% alcohol and two changes of 100% alcohol quickly. Lastly, the slides were cleared with two changes of xylene for 3 min each and coverslipped. The VVG staining displayed stained elastic fibers blue-black to black, nuclei blue to black, collagen red, while other tissue elements or background were yellow on observation with a light microscope.

### 2.4. Immunohistochemical Staining for CD68

For immunohistochemical staining procedures, the slides were initially deparaffinized and hydrated as described above. Heat-induced epitope retrieval using citrate buffer was performed for antigen retrieval (20 min at pH 6.0). Then, the slides were incubated with normal serum for 20 min (37 °C) to block nonspecific staining. After blocking, the slides were incubated with monoclonal antibody mouse anti-human CD68 MCA5709, Clone KP-1 IgG (1:50) from BIO-RAD (Hercules, CA, USA) in 10 mM sodium phosphatase, pH 7.2–7.4 for 30 min (37 °C). Afterwards, the slides were incubated using the biotinylated Vectastain Universal ABC-AP kit AK-5200 for 30 min (37 °C) as a secondary antibody. For visualization of the staining, the slides were incubated with Vector Red (Vector Labs, Burlingame, CA, USA) for 30 min (37 °C). Tris-buffered saline was used as a washing solution in all the immunostaining steps mentioned above. Subsequently, the slides were incubated for 10–15 min in alkaline phosphatase substrate solution, and then washed with deionized water for 5 min. Lastly, the slides were counterstained with Harris hematoxylin for 1–2 min, dehydrated, cleared, and mounted (Entellan, Merck Darmstadt, Germany) immediately. Positive CD68 staining was indicated by a red color, while negative CD68 staining was indicated by colorless areas. One minute hematoxylin was used as a counterstain, showing a blue color in the nuclei by means of a light microscope.

### 2.5. Analysis of Plaque Severity and Extent

Plaque severity and extent were measured from the VVG-stained slides. Plaque severity was determined using a well-established protocol for specifying a grade from I–III as initial lesions and IV to VI as an advanced lesion (American Heart Association Grade) [15,16]. The grade I lesions contain atherogenic lipoprotein (LDL) associated with an increase in macrophage infiltration and the formation of macrophages filled with lipid droplets (foam cells) in the arterial intima. This lesion usually occurred in arteries with adaptive thickening of the intima, which is normally very thin and represents adaptations to local mechanical forces. Grade II lesions consist of macrophage foam cells and smooth muscle cells containing lipid droplets designated as fatty streaks. Grade III lesions are characterized by pools of extracellular lipid droplets and particles in addition to all the components of grade II lesions. Grade IV lesions are characterized by a larger, united, and disruptive core of extracellular lipids known as the lipid core or atheroma. Grade V lesions have a lipid core, in which prominent new fibrous connective tissue has formed and is referred to as fibroatheroma. Some grade V lesions are also largely calcified. Lastly, grade VI lesions represent grade V lesions with disruptions of the lesion surface, hematoma, or hemorrhaging. This type may also be referred to as complicated lesions [18].

The plaque severity scores for each monkey were determined as the maximum severity grade in each CCA and BIF. We chose the maximum grade because the clinical event of cardiovascular disease, especially coronary artery disease, relates to its most complicated lesion [21]. Plaque extent was presented as the intimal area (IA), internal elastic lamina length (IELL), and maximum intimal thickness (MXIT) of the carotid artery. In each animal, the average of IELL, IA, and MXIT was calculated for both CCA and BIF.

### 2.6. Data and Statistical Analysis

Plaque severity of the carotid artery was analyzed descriptively. The extent of the plaque (IA, IELL, and MXIT) was analyzed quantitatively using Image-Pro Plus Ver 9.1. (Media Cybernetics^®^, Rockville, MD, USA) and reported as mean +/− standard error. The CD68 immunostaining was analyzed quantitatively using Visio Pharm image analyses (VISIO PHARM^®^, Broomfield, CO, USA). One-way analysis of variance (ANOVA) (IBM SPSS) was used to assess parameter differences among the different groups (a level of 0.05 was used as the threshold for statistical significance). A Duncan test was used for post hoc comparisons when the ANOVA result was statistically significant.

## 3. Results

### 3.1. Atherosclerosis Risk Factors

The atherosclerotic risk factors, which comprise blood lipid profiles, blood glucose, and BMI of all the three groups of monkeys, have been previously reported [9]. The results for the nine euthanized monkeys included in the current study are provided in Table 2, showing that LDL but not TG was increased with the response to cholesterol (hypo, intermediate, and hyper-responsive groups). The value of TG and Glu were highest in the intermediate group compared to the other groups, while BMI was the smallest. However, the BMI of monkeys from all the groups was under 30 kg/m^2^, indicating that they did not develop obesity. The blood glucose of monkeys in all of the groups was also in normal ranges.

### 3.2. Plaque Severity

**Figure 1:**
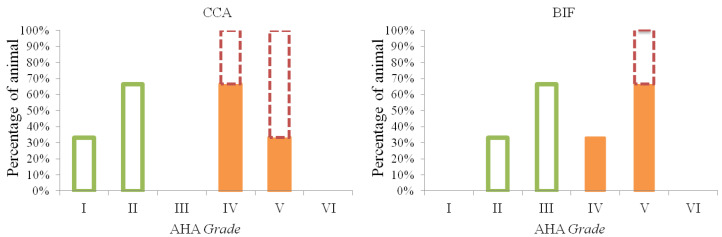
Different grades of atherosclerotic lesion on the common carotid arteries (CCA) and carotid bifurcation (BIF). In the hyporesponsive group (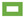), lesion grades I and II occurred on the CCA, whereas lesion grades II and III were found on the BIF. Lesion grades IV and V were observed in the intermediate group (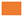) and hyper-responsive group (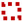). Grade V was more prevalence on the BIF compared to the CCA.

The monkeys receiving the IPB-1 atherogenic diet displayed different grades of atherosclerosis in the CCA and BIF (Figure 1). Advanced atherosclerosis plaques, grades IV and V, were presented by six animals out of nine animals, and initial atherosclerosis lesions (grade I–III) were presented by three out of nine animals. In the hyporesponsive group, atherosclerosis grade II was most common plaque observed on the CCA. In the intermediately responsive group, 66.7% monkeys had grade IV and 33.3% had grade V. Additionally, in the hyper-responsive group, atherosclerosis occurred mostly at grade V. These findings indicate that the hyper-responsive group presents the most severe grades for atherosclerotic lesions on the CCA compared to the other groups.

On the BIF, a maximum grade of III was observed in the hyporesponsive group. In the intermediately responsive group, 33.3% of the animals had a grade of IV, and 66.7 had a grade of V. In the hyper-responsive group, all the animals displayed a grade of V. None of the CCA and BIF sections showed an atherosclerosis lesion grade of VI. These results indicate that the atherosclerosis grade induced by IPB-1 atherogenic diet in the Indonesian cynomolgus monkey is more severe on the BIF compared to the CCA.

**Figure 2:**
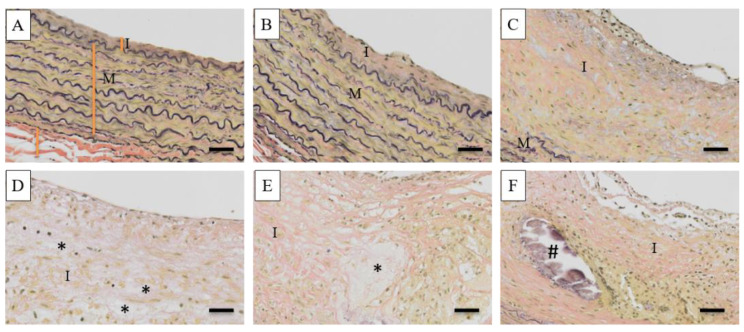
Histopathological stage of the atherosclerotic lesion in the carotid artery (Verhoef Van Giesson staining). (**A**) Normal artery showing a thin tunica intima (I) and thick tunica media (M). (**B**,**C**) The initial stage of the atherosclerotic lesion shows infiltration of macrophages, as well as increasing foam cells and smooth muscle. (**D**) Atheroma displays thickening of the intima with a few spots of necrosis. (**E**,**F**) Fibroatheroma shows a fibrous cap, the core of the necrosis (*), and calcification (#). Scale bars = 20 μm.

**Figure 3:**
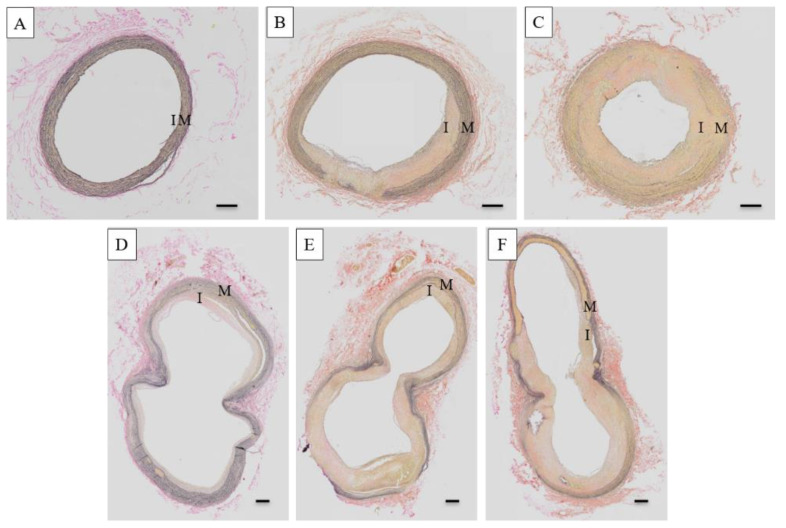
The atherosclerotic lesions in the common carotid arteries (CCA) (**A**–**C**) and bifurcation (BIF) (**D**–**F**) are increased with the response to cholesterol. Formation of atherosclerosis lesion increased the intimal (I) thickness and reduced elastic fibers in the media (M). No lesion was observed in the CCA of the hyper-responsive group (**A**). An eccentric lesion was developed in the CCA of the intermediate group (**B**), while a concentric lesion was developed in the CCA of the hyper-responsive group (**C**). A fatty streak lesion was noted in the BIF of the hyporesponsive group (**D**), whereas a fibroatheroma was found in the carotid bifurcation (BIF) of the intermediately and hyper-responsive groups (**E**,**F**). The most severe lesion in the CCA and BIF was observed in the hyper-responsive group (**C**,**F**). Scale bars = 200 μm.

The different histopathological stages of the atherosclerosis lesion on the carotid artery of the treated monkeys are shown in Figure 2, while photomicrographs of the CCA and BIF arteries from hypo-, intermediately, and hyper-responsive group monkeys are provided in Figure 3. The carotid artery wall consists of three layers, namely the tunica intima, media, and adventitia. The normal carotid artery is characterized by a thin intimal layer and a thick tunica media that comprise numerous elastic fibers (Figure 2A). At the beginning of atherogenesis, there is a recruitment of macrophages and an increased thickness of the intima (Figure 2B). The lesion’s progression continues to thicken the tunica intima of the artery by increasing foam cells (derived from macrophages), but also extracellular matrix content, and smooth muscle proliferation (called pre-atheroma) (Figure 2C). The advanced lesions are shown in Figure 2D–F. Necrosis, a fibrous cap, and calcification can be observed in the advanced lesions. Figure 3 shows that increasing the cholesterol responsiveness to the IPB-1 atherogenic diet exacerbated the formation of plaques in the carotid artery.

### 3.3. Plaque Extent

For the CCA, monkeys receiving an IPB-1 atherogenic diet had a mean IELL of 6.72 ± 0.10 mm, IA of 0.45 ± 0.06 mm^2^, and MXIT of 0.18 ± 0.018 mm. For the BIF, the mean IELL was of 13.93 ± 0.37 mm, IA of 2.23 ± 0.22 mm^2^, and MXIT of 0.46 ± 0.042 mm. The differences in IELL, IA, and MXIT for three groups of responsiveness are summarized in Table 3.

From the CCA data presented in Table 2, the IELL of the hyporesponsive group was the smallest compared to the other groups. However, the IELL did not differ between the intermediately and hyper-responsive groups (*p* = 0.382), indicating that IELL increased in the intermediately and hyper-responsive groups. The IA of the hyper-responsive group was the highest one, almost 20 times the IA of the hyporesponsive group. The smallest IA was found in the hyporesponsive group, while the IA of the intermediate group was between the hypo- and hyper-responsive groups. Similar findings were observed for MXIT, showing thicker plaques formed with higher responses to cholesterol.

For the BIF, there was no significant difference in IELL among the groups, indicating that the responsiveness and formation of lesions did not affect this parameter. Table 2 also shows that the smallest value for both IA and MXIT on BIF was in the hyporesponsive group. No significant difference was observed in the intermediately and hyper-responsive groups for IA and MXIT, suggesting that the intermediately responsive group was able to form an atherosclerotic lesion on BIF, similarly to the hyper-responsive group.

### 3.4. Immunohistochemical Staining for CD68

**Figure 4:**
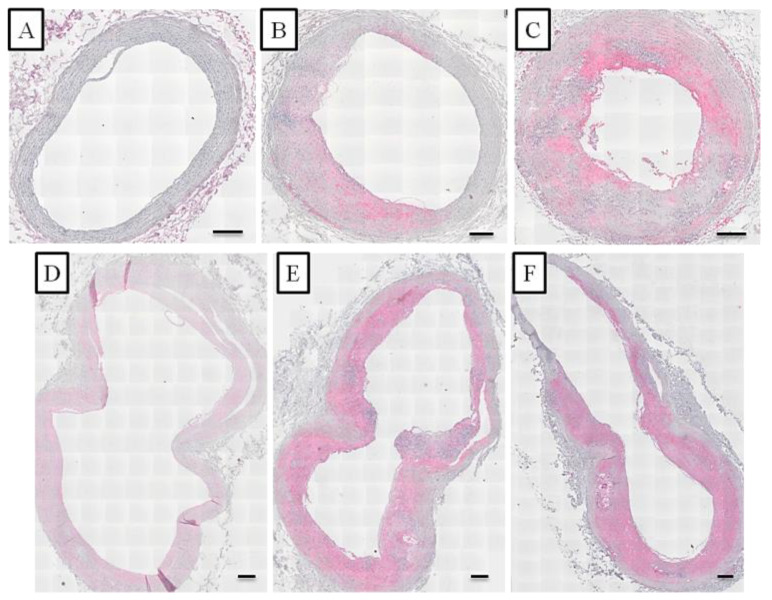
Immunohistochemical results of CD68 staining on the CCA (**A**–**C**) and BIF (**D**–**F**). The pink color indicates the positive CD68 visualized using vector red. The hyporesponsive group (**A**,**D**) had less prevalent CD68 staining, whereas the intermediately (**B**,**E**) and hyper-responsive groups (**C**,**F**) had the most prevalent CD68 positive staining. Scale bars = 200 μm.

The inflammatory marker CD68, which labels macrophages, was examined in this study. The percentages of CD68 staining of carotid arteries from macaques fed with the atherogenic IPB-1 are summarized in Table 4. For the CCA, approximately 1% of the examined arterial slides had a positive reaction for CD68 in the hyporesponsive group, but this proportion increased to 30% in the hyper-responsive group. For the BIF, positive CD68 was lowest in the hyporesponsive group, followed by the intermediately and the hyporesponsive groups. CD68-positive staining in the hyper-responsive group was found in approximately 60% of the arterial slides, showing less abundant macrophages in the CCA compared to the BIF. Photomicrographs of the carotid artery stained with IHC for CD68 are shown in Figure 4.

**Figure 5:**
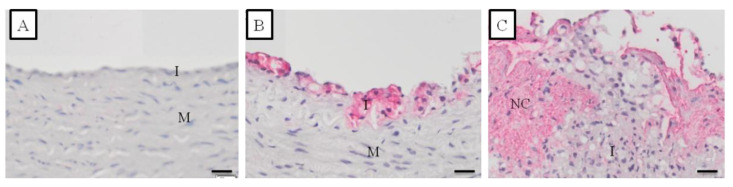
Distribution of CD68 staining at various stages of the atherosclerotic lesion formation in the carotid artery of the monkeys included in this study as detected by immunohistochemical (IHC) staining. The pink color indicated the positive CD68 visualized using vector red. (**A**) = normal artery; (**B**) = Fatty streak lesion (AHA grade II); (**C**) = Fibroatheroma lesion (AHA grade IV). I = tunica intima; M = tunica media; NC = necrotic core. Scale bars = 20 μm.

The staining for CD68 in the carotid artery of cynomolgus monkeys receiving the IPB-1 atherogenic diet had different distributions (Figure 5). Providing information on CD68 distribution supports the determination of arterial inflammation. Figure 5 shows no positive CD68 staining in the normal artery. In fatty streak lesions, positive CD68 was found in some areas at the top of the tunica intima, contrary to the tunica media. In atheroma and fibroatheroma lesions, macrophages were found at the surface of the intima, around the necrotic core, and at the “shoulder” or border area between the lesion and normal area. This result indicates that macrophages can enter the healthy tissue to expand the atherosclerosis lesion.

## 4. Discussion

The development of atherosclerotic lesions is influenced by many factors, such as genetics, age, sex, hypercholesterolemia, diabetes, cigarette smoking, and stress [22,23]. The IPB-1 atherogenic diet-induced atherosclerosis risk factors of hypercholesterolemia. Different groups (hypo-, intermediate, and hyper-responsive) had different blood lipid profiles (LDL, HDL, TG), Glu, and BMI. We found that LDL but not HDL increased with the response to cholesterol. The IPB-1 atherogenic diet contains egg yolks that can increase LDL [24] and high levels of carbohydrates that can decrease HDL [25]. Hyporesponsive monkeys in this study displayed the lowest TPC, LDL, TG, and Glu levels. A similar phenomenon was reported previously in cynomolgus monkeys receiving a Western diet [14]. Recently, in Indonesian cynomolgus monkeys, genetic diversity in the 3′UTR of LDLR gene was associated with low blood LDL levels [26] resulting in insufficient atherosclerotic lesions.

In this study, we observed an intermediately responsive group, whereas the Western diet only induced hypo- and hyper-responsive groups previously. The IPB-1 atherogenic diet has a moderate level of cholesterol, which aims to prevent death during a two-year period of treatment. Long-term feeding and very high TPC induced by the Western diet were reported to result in depression among cynomolgus monkeys [15]. Nevertheless, consumption of IPB-1 atherogenic diet for two years was able to induce advanced atherosclerosis lesions in six out of nine monkeys in the intermediately and hyper-responsive groups. This indicates that the IPB-1 diet can be used for long-term atherosclerosis research investigating treatments for atherosclerosis as well as the regression of atherosclerotic lesions, which requires advanced lesions as observed in this non-human primate model.

The atherosclerotic lesions observed in this study histologically showed the formation of foam cells, necrotic core, fibrous cap, and calcification. However, we did not find evidence for the formation of cholesterol cleft, thin cap, and rupture. During the treatment, we maintained cholesterol supply for the monkeys around 0.28–0.29 mg chol/Cal, which was lower than Western atherogenic diets comprising 0.35 mg chol/Cal. The reduced level of cholesterol could have prevented its interaction with complex atheroma lesions. Lowering dietary cholesterol contents by changing cholesterol intake may reduce the atherosclerosis risk of coronary heart disease considerably in highly responsible individuals [27]. These findings indicate that a IPB-1 atherogenic diet for two years cannot be used to induce a cynomolgus monkey model of full stenosis and atherosclerotic rupture.

The atherosclerosis lesions induced by the IPB-1 atherogenic diet in Indonesian cynomolgus monkeys were also more severe on BIF compared to CCA. Plaque severity and plaque extent of intermediately and hyper-responsive monkeys at the BIF are similar. This indicates that a slight increase in TPC could present the same risk of plaque formation on BIF. Severe atherosclerosis lesions on BIF are caused by complex fluid flow mechanics and geometric factors. High fluid flow raises the endothelial dysfunction of the artery and accelerates the atherogenesis on BIF [28,29]. Sinus enlargement of >1.2 times the CCA branch diameter is the most significant geometric risk factor for developing atherosclerosis in human BIF [30].

Plaque extent (IA and MXIT) on the CCA was lowest in the hyporesponsive group followed by the intermediately and hyper-responsive groups. Hypercholesterolemia caused by IPB-1 atherogenic diet thickens the tunica intima. Thickening of the tunica intima initiated by trapping and oxidation of LDL stimulates the endothelium to produce adhesion molecules and chemokines [31,32]. These inflammatory processes recruit specific infiltrating and proliferating cells, such as macrophages, lymphocytes, and smooth muscle cells in the tunica intima [8]. Consequently, raising cholesterol levels, especially LDL-cholesterol levels, increases the IA and MXIT of the artery.

Macrophages play important roles in the initiation of lesions, lesion expansion, and severe lesion formation, but also in the resolution and regression of atherosclerotic lesions [11]. The inflammation marker CD68 allows the visualization of the presence of macrophage cells in the artery. Macrophages are the most prevalent cells at the beginning of atherogenesis [13]. For the CCA, CD68 staining was very rare in the hyporesponsive group and represented around 33% of artery slides in the hyper-responsive group. Increasing responsiveness increased the positive staining for CD68 in the artery. For the BIF, CD68 staining was abundant in the hyporesponsive group and reached 50%–60% of the artery slides in the hyper-responsive group. The high CD68 showed high inflammation, which adversely affects the severity of atherosclerosis [33].

The distribution of CD68 staining in the Indonesian cynomolgus monkey’s artery was similar to previous reports in humans. The CD68 marker in the human artery is located on the surface of the tunica intima, the necrotic core, and shoulder of the lesion [34]. In the carotid artery of the monkeys examined in this study, not all foam cells showed positive staining for CD68, probably because foam cells are also represented by smooth muscle cells [35]. From the necrotic core, macrophages enter the fibrous cap and release metalloproteinase that degrade the extracellular matrix. Degradation of the matrix makes the tissue brittle and easy to rupture [36].

## 5. Conclusions

From these results, we can conclude that feeding with a local ingredient atherogenic diet for two years can induce atherosclerotic lesions on the carotid arteries of Indonesian cynomolgus monkeys. Different stages of lesions were observed, based on the responsiveness of the monkeys to cholesterol. The hyporesponsive group did not develop proper atherosclerosis lesions in carotid arteries, indicating the importance of screening monkeys before use in atherosclerotic research. The intermediately and hyper-responsive groups developed severe atherosclerotic lesions in carotid arteries, both in the CCA and BIF. The lesions formed were similar to those observed in humans and included the distribution of macrophage cells in the lesion. These macrophages were found in the early atherosclerotic lesions and continued to expand their infiltration to the healthy arterial tissue in advanced lesions. In future studies, it will be necessary not only to explore the lesion stages but also to investigate certain molecular conditions in the artery, especially coronary arteries, together with the condition of the primary organ affected by this diet.

## Figures and Tables

**Table 1:** Differences between IPB-1 atherogenic diet and Western diet [7,9,14,16].

Macronutrient	Western Diet (Sources/Level)	IPB-1 Diet (Sources/Level)
Protein (animal)	Casein and lactalbumin: high	Fish meal: moderate
Protein (plant)	Wheat flour: low	Soya meal: moderate
Saturated fats	Butter: high	Coconut oil and beef tallow: moderate
Unsaturated fats	Apple sauce: low	Corn oil: normal
Carbohydrates	Sugar, wheat flour: high	Sugar, wheat flour: high
Cholesterol	Cholesterol: high (0.33–0.35 mg chol/Cal)	Egg yolk: moderate (0.28–0.29 mg chol/Cal)

chol = cholesterol.

**Table 2:** Atherosclerosis factors of nine monkeys fed an IPB-1 atherogenic diet showing differences in responses between the three groups.

Parameter	Hyporesponsive	Intermediately Responsive	Hyper-Responsive
TPC (mg/dL)	158.15 ± 9.25 ^a^	335.56 ± 24.63 ^b^	478.94 ± 17.57 ^c^
HDL (mg/dL)	56.33 ± 2.73 ^c^	32.61 ± 2.95 ^b^	32.08 ± 1.46 ^a^
LDL (mg/dL)	88.18 ± 9.83 ^a^	202.66 ± 22.39 ^b^	342.70 ± 14.91 ^c^
TG (mg/dL)	40.00 ± 2.78 ^a^	64.33 ± 5.020 ^c^	42.53 ± 3.75 ^b^
Glu (mg/dL)	44.78 ± 2.02 ^a^	66.83 ± 4.22 ^c^	54.92 ± 2.69 ^b^
BMI (mg/dL)	25.21 ± 0.73 ^b^	22.68 ± 0.47 ^a^	26.34 ± 0.41 ^c^

TPC = total plasma cholesterol; HDL = high-density lipoprotein; LDL = low-density lipoprotein; TG = triglyceride; Glu = blood glucose, BMI = body mass index. Different superscripts were significant at *p* < 0.05 (^a^ lowest value followed by ^b,c^ to compare between hypo-, intermediately, and hyper-responsive groups in a row).

**Table 3:** Plaque extent in the carotid artery induced by IPB-1 atherogenic diet.

Artery	Parameters	Hyporesponsive	Intermediately Responsive	Hyper-Responsive
CCA	IELL (mm)	6.291 ± 0.123 ^a^	6.828 ± 0.128 ^b^	7.035 ± 0.225 ^b^
	IA (mm^2^)	0.045 ± 0.007 ^a^	0.473 ± 0.053 ^b^	0.821 ± 0.109 ^c^
	MXIT (mm)	0.034 ± 0.006 ^a^	0.216 ± 0.019 ^b^	0.305 ± 0.020 ^c^
BIF	IELL (mm)	13.959 ± 0.138 ^a^	13.630 ± 0.460 ^a^	14.212 ± 1.067 ^a^
	IA (mm^2^)	1.266 ± 0.302 ^a^	2.527 ± 0.283 ^b^	2.920 ± 0.201 ^b^
	MXIT (mm)	0.307 ± 0.074 ^a^	0.486 ± 0.051 ^b^	0.597 ± 0.034 ^b^

CCA = common carotid artery; BIF = carotid bifurcation; IELL = internal elastic lamina length; IA = intimal area; MXIT = maximum intimal thickness. Different superscripts were significant at *p* < 0.05. ^a^ lowest value followed by superscripts ^b,c^ to compare between hypo-, intermediately, and hyper-responsive groups in a row.

**Table 4:** Mean percentages of CD68-positive (%) staining on the carotid artery.

Artery	Hyporesponsive	Intermediately Responsive	Hyper-Responsive
CCA right	1.94 ± 1.11 ^a^	10.15 ± 1.97 ^b^	29.95 ± 4.25 ^c^
CCA left	1.08 ± 0.25 ^a^	14.26 ± 2.79 ^b^	33.35 ± 4.40 ^c^
BIF right	10.70 ± 4.01 ^a^	46.47 ± 5.64 ^b^	50.09 ± 6.25 ^b^
BIF left	16.67 ± 4.83 ^a^	44,10 ± 6.23 ^b^	60.89 ± 7.87 ^c^

CCA = common carotid artery; BIF = carotid bifurcation. Different superscripts were significant at *p* < 0.05. ^a^ lowest value followed by superscripts ^b,c^ to compare between hypo-, intermediately, and hyper-responsive groups in a row.

## Data Availability

The data presented in this study are available upon reasonable request to the corresponding author.
